# Feeling the Beat: Temporal Predictability is Associated with Ongoing Changes in Music-Induced Pleasantness

**DOI:** 10.5334/joc.286

**Published:** 2023-07-04

**Authors:** Neomi Singer, Nori Jacoby, Talma Hendler, Roni Granot

**Affiliations:** 1 Sagol Brain Institute and department of Neurology, Tel Aviv Sourasky Medical Center, Tel Aviv, Israel; 2 Sagol school of Neuroscience, Tel-Aviv University, Tel Aviv, Israel; 3 School of Psychological Science, Tel-Aviv University, Tel Aviv, Israel; 4 Max Planck Institute for Empirical Aesthetics, Frankfurt am Main, Germany; 5 Sagol Brain Institute, Tel Aviv Sourasky Medical Center, Tel Aviv, Israel; 6 Sackler School of Medicine, Tel-Aviv University, Tel Aviv, Israel; 7 Musicology Department, Hebrew University of Jerusalem, Jerusalem, Israel

**Keywords:** music, emotion, temporal-regularity, predictive-coding, arousal and valence

## Abstract

Music is a complex phenomenon that elicits a range of emotional responses, influenced by numerous variables, such as rhythm, melody and harmony. One interesting aspect of music is listeners’ ability to predict its continuation as it unfolds – an inherent attribute hypothesized to contribute to our emotional response to music. In this study, we investigated this link by examining the relationship between temporal predictability – the ability to predict the timing of the next event – and the ongoing changes in music-induced pleasantness. Temporal predictability was operationalized as the degree to which taps of 20 musically trained participants, who tapped to the beat along three naturalistic and highly contrastive musical pieces, were aligned. We then examined the degree to which this measure could explain the ongoing emotional experience, as reflected in continuous measures of arousal and valence, in a separate group of 40 participants that listened to these pieces. Our findings reveal a positive correlation between fluctuations in reported valence and temporal predictability, even when controlling for a set of other musical features, in four out of five musical sections. The only exception being a lyrical slow section. These findings were further supported by a large online database of annotated musical emotions (n = 1780 songs), where a consistent and robust correlation between valence ratings and an automatically extracted feature of pulse clarity was demonstrated. Overall, our findings shed light on the significance of temporal predictability as a contributing factor to the hedonic experience of music, especially within the tempo range of salient beat perception.

## Introduction

Emotions are a complex evolving composition of states-of-mind. In other words, an emotional experience does not simply switch on or off, rather it constantly emerges and changes in reaction to particular triggers in the internal or external environment. Music is a universally acknowledged robust emotion-provoking stimulus with a distinct temporal structure, therefore well-suited for investigating the unfolding emotional experience and its underpinnings. Indeed, previous studies that measured the ongoing emotional responses to music demonstrated that the emotions induced by music dynamically vary in time in close correspondence with the unfolding of music (Egermann, Pearce, Wiggins, & McAdams, 2013; Fredrickson, 1999; Grewe, Nagel, Kopiez, & Altenmüller, 2007; Krumhansl, 1997; Madsen, 1998; Schubert, 2004; Schubert & Dunsmuir, 1999; Timmers, Marolt, Camurri, & Volpe, 2006). In a comprehensive review of the literature, adjoining and expanding data and various theoretical accounts (e.g., Scherer & Zentner, 2001), Juslin and colleagues (2013; 2010) suggested that several complementary mechanisms may be involved in our emotional response to music. In this work, they outlined eight possible psychological mechanisms through which music may induce emotion (acronymed the BRECVEMA model). Among these proposed mechanisms, those of *musical expectancy* and *rhythmic entrainment*, directly relate to the dynamicity of music and highlight the importance of the momentarily divergence from- or generation of- expected patterns in music.

The ability to predict sensory information in time is fundamental for successful and adaptive interaction with the environment. Recent theoretical frameworks suggest that such predictive capacity constitutes a fundamental functional principle of the ‘proactive’ brain (Bar, 2009) whereby the brain constantly generates predictions about the upcoming input for perception (Friston, 2005) and control of action (Friston, FitzGerald, Rigoli, Schwartenbeck, & Pezzulo, 2017). The importance of predictability to our adaptive perception and behavior implies that it has hedonic value on its own. Indeed, evidence suggests that (un)predictability, especially in the temporal domain is associated with distinct affective responses. For example, in conditioning studies, it is well-established that unpredictable delivery of aversive events results in heightened anxiety-like behavior as compared to predictable ones (Grillon, Baas, Lissek, Smith, & Milstein, 2004). Remarkably, even neutral stimuli, when delivered in a temporally unpredictable fashion, promote anxiety-like behaviors (Herry et al., 2007; Jackson, Nelson, & Proudfit, 2015; Parisi, Hajcak, Aneziris, & Nelson, 2017), bias towards negatively valenced interpretations for ambiguous situations (Davis, Neta, Kim, Moran, & Whalen, 2016) and elicit heightened amygdala activation (Herry et al., 2007; Koppe et al., 2014). Complementarily, it has been shown that emotionally neutral stimuli that have a predictive value (infused by means of associative learning) are preferred and rated as more pleasant than those with a weaker predictive value (Trapp, Shenhav, Bitzer, & Bar, 2015).

As music generates clear expectations, especially in the temporal domain via its rhythmic properties (Vuust & Kringelbach, 2010), it is plausible to assume that variations in such predictability while listening affect the manner by which it is experienced. Indeed, musical expectations and their violation have been long conceptualized to be associated with music-induced emotions, in particular their hedonic aspect (Berlyne, 1971; Huron, 2008; Juslin, 2013; Koelsch, Vuust, & Friston, 2019; Meyer, 2008; Salimpoor, Zald, Zatorre, Dagher, & McIntosh, 2015; Vuust & Witek, 2014). Evidence from recent years supports these theoretical proposals, by revealing that music-induced emotions are elicited during moments of unpredicted events of ‘musical surprises’ (Brattico, Jacobsen, De Baene, Glerean, & Tervaniemi, 2010; Egermann et al., 2013; Shany et al., 2019; Steinbeis, Koelsch, & Sloboda, 2006), and vary with the levels of harmonic, melodic, or rhythmic complexity of the music (Bonin, Trainor, Belyk, & Andrews, 2016; Cheung et al., 2019; Sauvé, Sayed, Dean, & Pearce, 2018). Evidence further suggests that such a relationship between the complexity of music and the liking or preference of music may follow an inverted U-shape function, with a “sweet spot” at medium levels of complexity (Bianco, Gold, Johnson, & Penhune, 2019; Chmiel & Schubert, 2017; Gold, Pearce, Mas-Herrero, Dagher, & Zatorre, 2019; Heyduk, 1975; Matthews, Witek, Heggli, Penhune, & Vuust, 2019; Stupacher, Wrede, & Vuust, 2022; Witek, Clarke, Wallentin, Kringelbach, & Vuust, 2014), as anticipated from optimal complexity models of preference (Berlyne, 1971; Walker, 1972).

With a particular focus on the temporal domain, previous studies that alluded to the role of temporal expectancy in music-induced emotions showed that they vary with musical attributes related to temporal prediction violation, as syncopation (Matthews et al., 2019; Stupacher et al., 2022; Witek et al., 2014), or to temporal regularity, as extracted from the acoustic signal; i.e., pulse clarity (Eerola, 2011; Trost, Frühholz, Cochrane, Cojan, & Vuilleumier, 2015). Another line of investigation alluded to such a link by showing that the affective responses to music, on the scales of valence and arousal, vary as a function of the computationally modeled temporal “unexpectedness” of each note (Sauvé et al., 2018). Yet, while these findings support the notion that temporal regularities play a role in music-induced emotions, it remains to be directly examined if this aspect relates to one’s ability to predict the next event using an index that represents the cognitive percept of active inference. Additionally, though the temporal dimension is inherent to the definition of music and to the emotional experience it triggers, only a limited number of studies have incorporated the issue of dynamism by modeling the moment to moment contribution of musical attributes to the emotional experience using temporally continuous measurements (Egermann et al., 2013; Fredrickson, 1999; Grewe et al., 2007; Krumhansl, 1997; Madsen, 1998; Sauvé et al., 2018; Schubert, 2004; Schubert & Dunsmuir, 1999; Timmers et al., 2006). The use of dynamic measurements is especially important when considering the role of essentially temporal phenomena, as temporal predictability, in eliciting emotional experiences.

In the current study, we address these issues by assessing the contribution of temporal predictability to music-induced emotions as they unfold during listening to naturalistic music. We used a rich behavioral dataset that was part of a previous fMRI study exploring various aspects of the ongoing affective response to music (Singer et al., 2016). This dataset contained the continuous affective responses to three naturalistic musical pieces. Responses included subjective ratings on the scales of valence and arousal. To index the aspect of temporal predictability in music – a high-level cognitive percept that cannot be accurately indexed in by automatic music-information-retrieval approaches – we used experts-based annotation approach. This approach is based on the beat-tapping patterns of a different group of 20 musically trained participants. Temporal predictability was operationally defined as the extent of tapping coherence across the different tappers, under the assumption that the more predictable the next beat, the more participants will synchronize their tap to it (within a narrow range of 100 ms). To gain a wider understanding into the importance of temporal predictability in explaining the affective experience, we analyzed the data while taking into account additional musical dimensions known to contribute to the ongoing experience, such as pitch, tempo and loudness. Finally, in order to generalize to a wider set of musical materials, we examined a large dataset of dynamic annotation of musical emotions (valence and arousal; Supplementary file) (Aljanaki et al., 2017) and assessed the link between the subjective reports and pulse clarity – an automatically extracted index that serves as a proxy of predictability. This measure relies on the analysis of regularities in the sound itself, rather than on the tapping-based percept. We hypothesized that there will be a high correspondence between temporal predictability and music-induced emotions, particularly with their hedonic aspect (i.e., pleasantness), even when accounting for additional musical features. Based on growing body of evidence that points to an inverted u-shape between relationship music-induced pleasure and melodic (Gold, Pearce, et al., 2019), harmonic (Matthews et al., 2019) or rhythmic (Stupacher et al., 2022; Witek et al., 2014) complexity, we further expected the relation between music-induced pleasantness and temporal predictability to follow an inverted u-shape function across this large set of songs.

## Methods

### Participants

Forty healthy participants (22 females) between the ages of 19 and 33 (M = 25.5 ± 3.6 years) participated in the experiment, which included listening to three musical pieces, termed hereafter Ligeti, Glass and Mussorgsky (see details below). This sample size falls well within the norm of fMRI and psychophysiological studies that focused on the responses to fairly long naturalistic stimuli such as films (Raz et al., 2012), stories (Yeshurun et al., 2017) and music (e.g., Alluri et al., 2017; Coutinho & Cangelosi, 2011). The participants had no known history of neurological or psychiatric disorder and had a wide range of musical training, from none to 22 years of experience (*M*_experience_ = 5.39 ± 5.77 years). All participants provided written informed consent according to the Tel Aviv Sourasky Medical Center institutional review board (IRB) committee guidelines prior to the experiment.

### General Procedure and Data Acquisition

In the current investigation we used behavioral data that was collected as part of a large scale fMRI experiment exploring the neural underpinnings of the ongoing musical emotional experience and is described in detail elsewhere (Shany et al., 2019; Singer et al., 2016). The experiment was approved by the Tel Aviv Sourasky Medical Center institutional review board (IRB). The data presented here addresses a particular question related to the role of temporal predictability and is independent from our previous publications. Briefly, following the fMRI scan, the participants were requested to listen to the three musical pieces and to provide continuous online reports of their felt emotional experience on a two dimensional scale of valence and arousal using the EMuJoy software (Nagel, Kopiez, Grewe, & Altenmuller, 2007). During the rating session, participants were seated in a quiet room and presented with the musical pieces through Sennheiser HD 202 headphones (18–18000 Hz, sound level adjusted by the listener). Each music presentation was preceded and followed by a 30 seconds (S) epoch of silence. At the end of each rating session, the participants were further requested to fill out a detailed questionnaire about their listening experience: the 45 items of the Geneva Emotional Musical Scale, translated into Hebrew (GEMS-45; Zentner, Grandjean, & Scherer, 2008). Participants were additionally requested to rate how well they knew the piece and how much they liked it. Ratings were obtained with 5-point Likert scales ranging from 1 (“not at all”) to 5 (“very much”).

### Musical Stimuli

The musical stimuli consisted of three recorded piano pieces: (1) Ricercatas no. 1 & 2 from *Musica Ricercata* by György Ligeti (2:57 min and 4:53 min, respectively), (2) *The Hours* by Phillip Glass (piano arrangement: 7:03 min). Pieces 1 and 2 were recorded in-house using a Yamaha Disklavier upright piano and performed by Rotem Luz. (3) *Night on Bald Mountai*n by Modest Mussorgsky [Piano version: 10:57 min, performance by Boris Berezovsky, Teldec (Warner Classics), 1996]. Description of the pieces appears in Shany et al. (2019). Since the study focused on a limited number of pieces, these were chosen so as to represent many important contrasts found in musical pieces: tonal (Glass, Mussorgsky) vs. atonal (Ligeti); melody vs. harmony; regularity vs. irregularity in a host of temporal aspects (beat, meter, accentuation, grouping, tempo); rich vs. poor pitch content; and various textures. These are all presented within a generally clear structure of phrases and sections with literal or varied repetitions of melodic, harmonic and rhythmic patterns and phrases. These pieces were shown in a pre-test (*n* = 17) to elicit qualitatively different affective experiences in terms of their valence, which was relatively positive in Glass (*M* = .25 ± .31), moderate in Mussorgksy (*M* = .02 ± .29) and negative in Ligeti (*M* = –.43 ± .30). To account for each musical context separately, we treated both of Ligeti’s Ricercatas as separate pieces and distinguished between two qualitatively different sub-sections of Mussorgsky’s piece: part A (0:00–7:32) and B (7:33–10:57). To note, although the musical pieces were used as soundtracks for famous film features [“eyes wide shut” (Kubrick, 1999) for Ligeti, “the hours” for Glass (Daldry, 2002), “Fantasia” for Mussorgsky (Disney, 1940)], their familiarity within our participants’ pool was low (median ratings of familiarity <= 2, corresponding to the labeling of “to a little extent”) and did not differ across the pieces (Kruskal Wallis test (H2, N = 106) = 2.3, p = .32, Ligeti: *median* = 2, *n* = 37; Glass: *median* = 1, *n* = 36, Mussorgsky: *median* = 2, *n* = 33).

### Data Preprocessing and Analysis

#### Behavioral Indices

The individual ratings of valence and arousal, as indicated by the participant’s position on a two-dimensional affect space of valence and arousal, ranging from –1 to 1, were interpolated to obtain an evenly spaced time course at the resolution of 1 Hz. In some instances, ratings were not gathered due to technical difficulties (Ligeti, N = 1; Glass, N = 2; Mussorgsky, N = 3). Participants that presented a markedly distinct rating pattern on either valence or arousal scales were considered as outliers and removed from further analysis. The similarity of ratings across participants was assessed per musical piece by estimating the correlation between each participant’s rating and the average ratings of the rest of the participants (i.e., inter-subject correlation). Participants’ ratings whose correlation with the average rating was 2 standard deviations lower than the group average were considered markedly distinct and removed from further analysis (Ligeti: N = 2; Glass: N = 2; Mussorgsky: N = 3). Following this elimination procedure, analyses included data from 37 participants for Ligeti (*M* = 25.85 ± 3.47 years, 20 females), 36 participants for Glass (*M* = 25.87 ± 3.55 years, 20 females) and 34 participants for Mussorgsky (*M* = 25.44 ± 3.6 years, 17 females).

#### Annotation of Temporal Predictability

**Figure 1:**
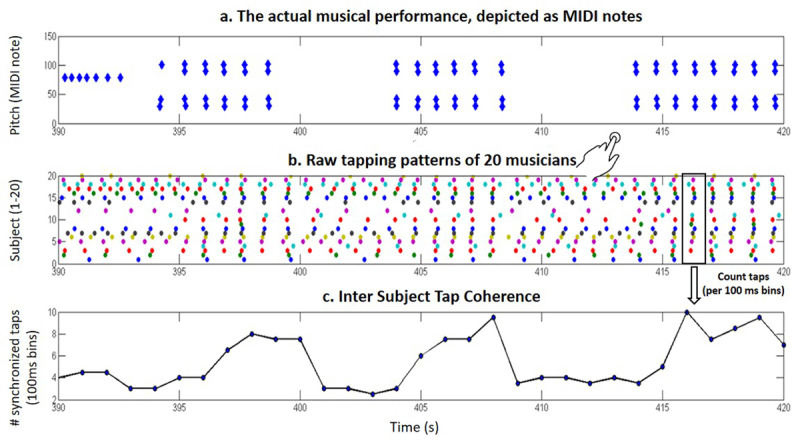
**Indexing of temporal predictability using tapping data.** Example for the indexing approach is given for a 30 s long section from Ligeti’s 2^nd^ Ricercata. **a.** *The actual physical stimulus* is graphically presented by indicating the MIDI notes played in each second. **b.** *Tapping pattern of twenty musically-trained participants* who were requested to tap along the beat. These data were used to calculate **c.** *Inter-Subject-Tapping-Coherence*: temporal predictability was operationally defined as the extent of synchronization of taps across the different experts, under the assumption that the better predicted the next beat the more participants will tap to it (within a narrow range of 100 ms). This index was assessed per second as the maximal number of synchronized taps across participants within that second.

In the current study, we aimed to create a continuous index that captures ongoing fluctuations in temporal predictions, which rely on the percept of the musical beat. Beat perception is a high-level cognitive percept that cannot be directly extracted using music information retrieval approaches that use automated signal processing based mainly on one or more acoustic features (e.g., Lartillot, Eerola, Toiviainen, & Fornari, 2008; McKinney, Moelants, Davies, & Klapuri, 2007). Even when applying state-of-the art multi-model approaches, the models’ output does not match human performance (except only under certain conditions), and their performance is significantly influenced by the musical style (Böck, Krebs, & Widmer, 2014) or beat interpretations of the rhythm (Miguel, Sigman, & Fernandez Slezak, 2020). We therefore, turned to experts-based annotation to index this percept (Figure 1). Specifically, temporal predictability was indexed using an annotation approach that is based on the beat tapping patterns of an independent sample of 20 musically trained participants, who regularly played an instrument for at least seven years (*M*_age_ = 26.15 ± 5.04; 8 females; *M*_years of playing_ = 15.2, SD = 5.17). This annotation experiment was conducted at the Hebrew University of Jerusalem, and received an IRB approval from this institution. After signing an informed consent, each participant was first asked to tap to the beat using the “Sonic Visualizer” (screen switched off) as an interface (version 1.7.2; Cannam, Landone, & Sandler, 2010). The “Sonic Visualizer” application allows for the simultaneous playback of music and recording of perceived beats using taps. Participants tapped along with the music using the “;” key on the keyboard, with each key press indicating the timing of a perceived beat. The Sonic Visualizer application recorded the timing of the taps in relation to the music. To minimize any bias from visual cues, the visual display (i.e., the monitor) was turned off once the tapping began, ensuring that participants relied solely on their auditory perception to tap along with the beat.

Tapping events with an inter-tap-interval shorter than 100 ms or longer than 3000 ms were considered implausible and removed from analysis. This procedure yielded a continuous, human-based “beat tracking” of each piece (Figure 1b). As we adopted a group-centered approach for the annotation, we first selected the datasets that presented fairly similar and consistent tapping patterns via a clustering approach. This was achieved by estimating the similarity (i.e., Pearson correlation) between the time series of inter-tapping-intervals (the time interval between successive taps in each second). Then, we applied a clustering algorithm (*cluster.m* function running on Matlab) to partition this similarity matrix into two clusters (high and low similarity). Finally, we selected the group that presented the higher within cluster consistency (Glass: N = 11, Ligeti: Ricercata 1: N = 18; Ricercata 2: N =19, Mussorgsky: part A: N = 19; part B: N = 18). The goal of this step was to both remove outliers and to prevent potential confounds that could arise from different perceptions of the meter or tactus at various metric levels (Martens, 2011). For example, Supplementary Figure S2 illustrates two groups with distinct patterns of beat-tapping along the Glass piece, which transitions from triple to duple meter. Importantly, post-hoc analysis using the entire dataset confirmed that this methodological decision did not significantly alter the reported results (see details in Supplementary Table S3). The resulting consistent subset of tapping data were then used for extracting an index reflecting temporal predictability as the *Inter-subject-tapping-coherence* (Figure 1c). Specifically, temporal predictability was operationally defined as the extent of synchronization of taps across the different tappers, under the assumption that the more predictable the next beat is, more tappers will tap to it within a narrow range of 100 ms. To extract this index, we counted how many tappers tapped within bins of 100 ms (with an overlap of 50 ms). Point estimates (per second) of the Inter-subject tapping coherence were obtained per second as follows:


IST{C_t} = \max ({S_{{\Delta _b}}})

For each 100 ms long time bin Δ*_b_* within a given time point *t*, we counted the number of subjects *S* that tapped within this narrow bin. Then, for each time point t, the maximal value of *S* out of time windows *w* \mathop \int \nolimits_w^1 {\Delta _{b\;}} was selected as representing *ISTC*_t_ – the inter-subject-tapping-coherence in a given second.

#### Annotation of Additional Musical Features

To gain a more accurate picture of the specific role of temporal predictability, we extracted additional musical features that are known to have a role in providing emotional cues in music (Coutinho & Cangelosi, 2011; Eerola, 2011; Eerola, Lartillot, & Toiviainen, 2009; Gabrielsson & Lindström, 2010; Schubert, 2004). The rationale for this step was two-fold: (1) to characterize the novel annotation by comparing it to other previously reported similar features (Lartillot, Eerola, et al., 2008) (2) to partial out the role of other musical features known to affect music-induced emotions by applying regression analyses. Specifically, we extracted from the sound files several low-level and high-level features that capture the fluctuations in loudness, timbre, pitch height and several temporal features. For example, *loudness* was assessed by extracting dynamic loudness using PsySound3 toolbox (Cabrera, Ferguson, & Schubert, 2007). *Pitch height* was estimated using two automatically extracted measures: height of the autocorrelation peak using Psysound3, and chromagram, using MIR toolbox (Lartillot, Toiviainen, & Eerola, 2008). *Timbre/Spectral content* was characterized by extracting spectral centroid and brightness using the MIR toolbox. These measures describe the prevalence among all or high-frequencies in the sound, respectively, for centroid and brightness. *Roughness* was additionally extracted using MIR toolbox as a proxy for momentary levels of sensory dissonance. Additional features, namely pulse clarity, event density, spectral flux, attack time, attack slope, spectral irregularity and tonal centers were extracted with MIR toolbox. These measures were complemented with two additional experts-based annotations: (1) *Tempo* – was extracted per second as the frequency of tapping, which was calculated as the one over median of inter-tap interval across tappers in that particular second. This index was multiplied by 60 to obtain a continuous assessment of beats per minute; (2) Musical surprises: an index that was used in our previous work to describe moments of expectancy violation in the music (Shany et al., 2019). This feature was indexed based on a second annotation session, which followed the tapping session, during which the experts heard each piece again and marked any musical event that sounded surprising to them. Surprises were assessed per second as the number of participants that pointed to a surprise in that particular second (for the full list of extracted features, see Supplementary Table S1).

### Statistical Analyses

Statistical analyses were performed with the SPSS 20 software statistical package, the statistical toolbox running on Matlab (2020b) and with custom code written with R (version 4.21) and running on RStudio (version 2022.07.2).

The association between the time series representing the musical features and the music-induced responses was assessed using a two-level random-effects non-parametric analysis approach. Specifically, first-level correlation or linear regression analysis was initially applied at the single subject level to assess the association between each individual response pattern (e.g., valence or arousal rating) and the annotated musical attribute (e.g., inter-subject-tapping-coherence). Then, the statistical significance of the resulting group correlation or regression coefficients was estimated using permutation testing based on phase-randomization (Honey, Thompson, Lerner, & Hasson, 2012). The inference using this approach is done by assessing the percentile location of the mean of correlation or regression coefficients (across the group) in relation to a null distribution of the mean correlation or regression coefficients. The null distribution of the mean coefficients was reconstructed by repeating 10,000 times by applying the same correlation or regression analysis, with the important exception that the phase of the time series representing the music-induced responses was first scrambled. This approach allows assessing the statistical likelihood of each observed correlation under the presence of autocorrelation as it leaves the power spectrum of the shuffled time series intact (Honey et al., 2012). To account for the possible delay between the musical features and the participants’ affective reaction (Bachorik et al., 2009; Schubert, 2013), a time delay of between 0 and 4 s between the time-series was applied. We used a two-level data-driven approach to select the exact timing of this delay. First, the regression analyses were performed by applying delays of 1-s increments between 0 and 4 s to the time-courses. The selected time lag, which was then used in all datasets for assessing the relationship between the ratings and musical features, was determined at the group level as the median of the optimal subject-specific time lags, which yielded the maximal regression coefficient in its absolute value.

In cases where first-level analysis was required (e.g., for assessing the correlation between different musical features), the statistical inference was done using the same phase-randomization-based approach (Honey et al., 2012). Here, the inference was done by assessing the percentile location of the obtained correlation coefficient in relation to a null distribution of correlation coefficients. To allow for stabilization of ratings – avoiding lower reliability at the beginning and end of continuous rating data (Bachorik et al., 2009; Schubert, 2013) – the first and last 30s of the rating time courses were removed from all analyses that include these data types.

#### Factor Analysis

To avoid redundancy and in order to identify distinct groups of musical features sharing common variance, we applied exploratory factor analysis using principle component analysis. Principal component analysis and orthogonal varimax rotation was conducted on 15 musical features capturing different perceptual attributes (loudness, timbre, pitch, tempo, etc., for a full list of features, see Supplementary Table S1) following Eerola (2011). This analysis was applied to z-scores of the different musical annotations, and across the three pieces, using IBM SPSS 20 software (IBM SPSS Statistics, IBM Corporation, Armonk, NY). The first nine resulting principal components were selected as they accounted for 90% of the variance in all pieces and used in further analyses. Factor profiles were determined based on the highest component loadings in the varimax rotated matrix above 0.6, and are presented in Supplementary Table S2. The factor scores were then calculated per component using regression (as implemented in SPSS) and were used to examine if the relationship between music-induced emotions and temporal predictability is evident when accounting for additional factors.

### Benchmark Dataset of Dynamic Annotation of Musical Emotions:

To support the observed findings and generalize them into a larger and more diverse musical sample using a different metric, we used the MediaEval Database for Emotional Analysis in Music (DEAM) – a large dataset containing dynamic annotations of valence and arousal for 1802 songs (Aljanaki et al., 2017). The database contains 1802 excerpts and full songs and their corresponding annotations of subjectively reported valence and arousal values both continuously and over the whole song, each acquired from 10 different participants using the Amazon Mechanical Turk (MTurk). Technical difficulties limited the analysis to 1780 out of 1802 songs. Each of the sound files were analyzed to extract pulse clarity using the MIR toolbox (Lartillot, Toiviainen, et al., 2008) running on Matlab. Pulse clarity is an automatically extracted measure that assesses “the ease of tapping to the beat” (Lartillot, Eerola, et al., 2008) – and was found to be correlated with the predictability index in our study (for details, see results section). Then, we assessed the association between the annotated pulse clarity and the affective annotation of each song; the average valence or arousal ratings per song. This association was assessed for the average continuous ratings per song (average value across the entire piece) using linear regression (lm function running on R). Given that the valence and arousal annotations are derived from the average ratings of ten different raters, we first assessed the inter-rater reliability of each song using the inter-group agreement index rWG, which was calculated using the rwg function from the *multilevel* package running on R. Songs with an rWG index lower than 0.7 were removed from the analyses (James, Demaree, & Wolf, 1984). The decision to use pulse clarity as an index of rhythmic complexity, instead of inter-subject tapping-coherence, was based on practical reasons, as collecting tapping data from experts for this large dataset of over a thousand songs was not feasible. Pulse clarity is an automatically extracted feature that is a proxy for how easily the beat is perceived, and it has been used in previous studies to index rhythmic complexity (Fujii & Schlaug, 2013; Stupacher et al., 2022). Correspondingly, we found a correlation between pulse clarity and tapping-coherence in the three pieces used in our study, as detailed in the Results section below.

## Results

### Characterization of the Tapping-Based Annotations of Temporal Predictability

**Figure 2:**
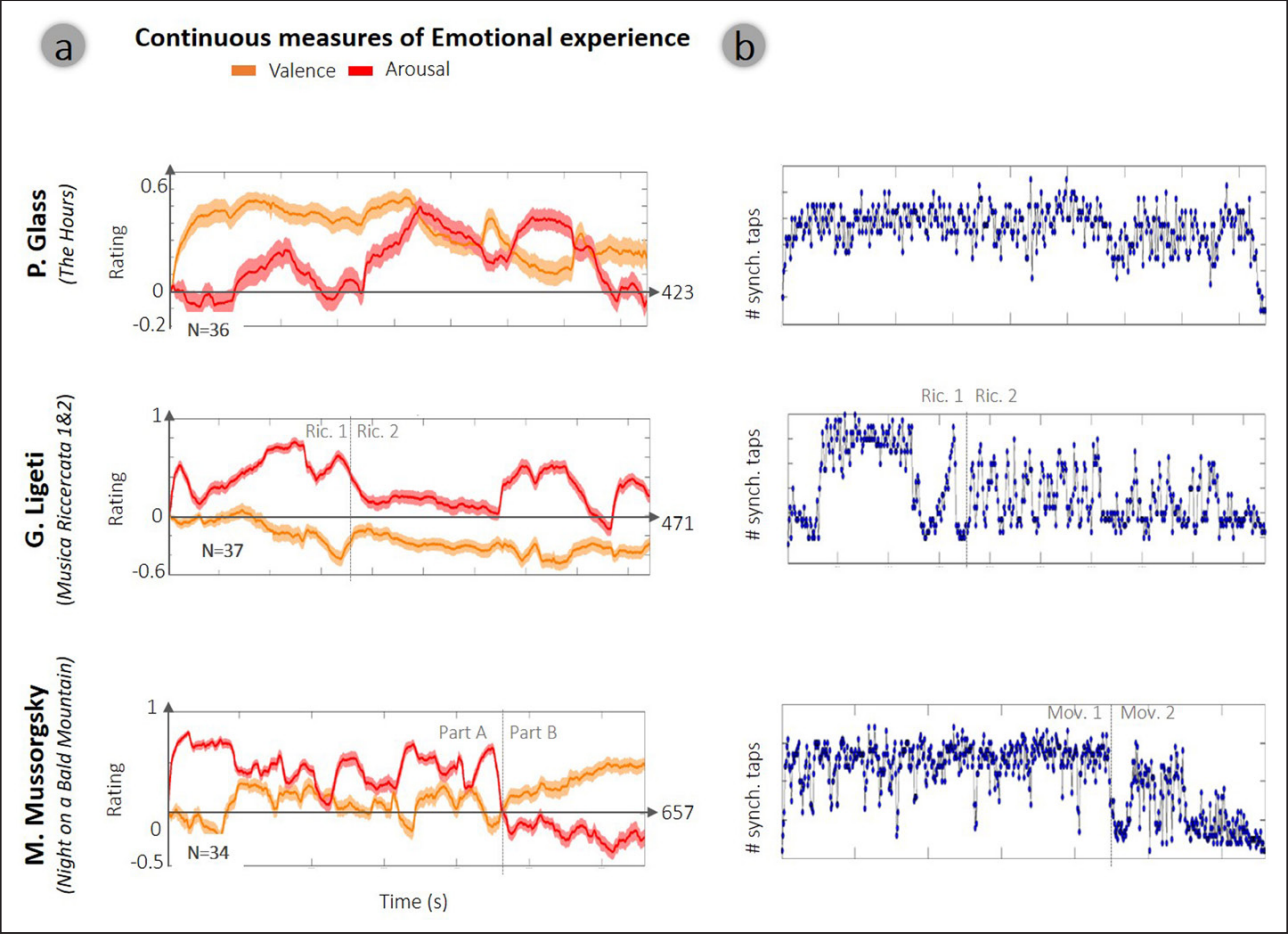
**Continuous reports of music-induced emotions and the corresponding indices of temporal predictability. (a)** mean intensity of the continuous reported music-induced experience on the dimensions of valence and arousal. Lines represent mean values of arousal and valence and thickness of shading represents 1 deviation from the mean (SEM). **(b)** Time series of the tapping based index denoting temporal predictability of *Inter-Subject-Tap-Coherence* per piece. Dashed lines indicate the point of transition between the two parts in Ligeti’s and Mussorgaky’s pieces.

As the focus of this study is to assess how temporal predictability in music is associated with music-induced emotions, we introduced a novel tapping based index: inter-subject-tapping-coherence (see methods for details). This index was depicted per second to provide a continuous index of predictability as the music unfolds (Figure 2). We compared this index to a set of automatically extracted features from the sound files that were designed to capture rhythm-related information: (1) pulse clarity (2) tempo (3) event density – all extracted with the MIR toolbox using a frame-based approach, with an analysis frame of 2 s and an overlap of 50% (Eerola, 2011) and shifted by 1 s. To identify robust associations, we only highlighted the features that were consistently and significantly correlated in all three pieces. As expected, the inter-subject tapping-coherence correlated with the index for pulse clarity in all pieces: (Ligeti, *r* = .69, *p* < .001; Glass *r* = .21, *p* = .04; Mussorgsky, *r* = .52, *p* < .001). To further characterize the tapping data, we extracted an index of tempo, calculated as the one over the median of the inter-tap interval per second and multiplied by 60 to obtain a continuous assessment of beats per minute. This measure nicely correlated with the automatically extracted MIR measures of event density (Ligeti, *r* = .36, *p* < .005; Glass *r* = .52, *p* < .0001; Mussorgsky: r = .29, *p* < .005) and an index of tempo extracted based on the automatic detection of onsets from the sound file and the subsequent calculation of one over the inter-onset interval (Ligeti: *r* = .54, *p* < .001; Glass: r = .39, *p* < .001; Mussorgsky: r = .55, *p* < .0001).

### Association between the Musical Emotional Experience and Temporal Predictability

We next turned to test the hypothesis that temporal predictability in music is associated with music-induced emotions as music unfolds by assessing the second-level correlation between the continuous reports (Figure 2a) and the inter-subject tapping-coherence (Figure 2b). The results of this analysis, which are detailed in Table 1, reveal that the reported *valence* was associated with temporal predictability in four out of the five musical segments examined: Glass’ piece, Ligeti’s 1^st^ and 2^nd^ Ricercatas and part A in Mussorgsky’s piece (*p* < .05, FDR corrected). Specifically, there was a significant positive correlation across subjects between the time-series of valence and inter-subject tapping-coherence. This indicates that during more temporally predictable moments, when inter-tapping coherence was high, participants tended to report experiencing higher levels of pleasantness. We further validated this observation by applying a paired t-test to compare between the average ratings during moments characterized with high vs. low temporal predictability (above the 75^th^ percentile or below the 25^th^ percentile of that index, respectively). Indeed, the average valence ratings in Glass’ piece, Ligeti’s 1^st^ and 2^nd^ Ricercatas and part A in Mussorgsky’s piece were significantly higher during moments of high temporal predictability than during moments of low temporal predictability, as indexed by inter-subject tapping coherence (*p* < .05, FDR corrected; see Table 1a). Arousal ratings, on the other hand, were not consistently correlated with inter-subject tapping-coherence across pieces, nor were found to differ between moments that are high vs. low in this index of temporal predictability across all pieces (Table 1b).

**Table 1:** Association between temporal predictability and behavioral responses to music: summary of correlation analyses and paired t-tests.


	A. VALENCE		

	MEAN *r*(SEM)	p-VALUE (BOOTSTRAP)	COMPARE HIGH VS. LOW

**Glass** (*n* = 36)	.12 (.02)	<.001***	*t*(35) = 4.42; *p* < .001***

**Ligeti, Ric. 1** (*n* = 37)	.23 (.07)	<.001***	*t*(36) = 3.86 *p* < .001***

**Ligeti, Ric. 2** (n = 37)	.04 (.02)	.05*	*t*(36) = 2.27; *p* = .0294*

**Mussorgsky, Part 1** (*n* = 34)	.08 (.01)	<.001***	*t*(33) = 2.97; *p* = .0055*

**Mussorgsky, Part 2** (*n* = 34)	–.24 (.04)	<.001***	*t*(33) = -5.53; *P* < .001***

	**B. AROUSAL**		

	**MEAN *r*(SEM)**	**p-VALUE (BOOTSTRAP)**	**COMPARE HIGH VS. LOW**

**Glass**(*n*=36)	–.02 (.02)	.54	*t*(35) = –1.73; *p* = .09

**Ligeti, Ric. 1** (*n*=37)	–.06 (.05)	.32	*t*(36) = –1.28; *p* = .21

**Ligeti, Ric. 2** (n=37)	–.05 (.02)	.006**	*t*(36) = –2.41; *p* = .02*

**Mussorgsky, Part 1**(*n* = 34)	–.02 (.02)	.24	*t*(33) = –.46; *p* = .65

**Mussorgsky, Part 2**(*n* = 34)	.35 (.03)	<.001***	*t*(33) = 7.95; *P* < .001***


*Note*: Averages and S.E.M of correlation coefficients between inter-subject tapping coherence and the ongoing fluctuations in reported a. valence or b. arousal per musical excerpt. The statistical significance, which was estimated using a phase randomization bootstrapping approach, is further indicated. T-values representing the result of a paired sample t-test for the comparison between the average ratings during moments of high vs. low moments temporal predictability are further provided. Effects corrected for multiple comparisons are highlighted in gray (FDR-corrected, p < .05). *Abbreviations*: Synch. = synchronization.

Together, these findings suggest that there is an association between temporal predictability and valence across distinct musical contexts, with the exception of the second part of Musssorgsky’s piece. One salient characteristic of this part, which may be related to this deviant observation, is its very slow tempo. Indeed, inspection of the distribution of the tapping-based tempo index, which is depicted per musical section in Supplementary Figure S1, revealed that the entire section falls outside the range for salient beat perception, between 80 and 160 beats per minute (Møller, Stupacher, Celma-Miralles, & Vuust, 2021), and is characterized by a slow tempo overall (*M*_bpm_ = 60.45).

### Association between temporal predictability and reported music-induced experience within the context of additional musical features

We next turned to examine whether temporal predictability still constitutes a significant factor that explains the ongoing affective experience when additional musical dimensions known to contribute to the ongoing experience, such as pitch, tempo and loudness (Coutinho & Cangelosi, 2011) are also taken into account. For that, we used a set of nine orthogonal principal components, which included, in addition to the inter-subject-tapping-coherence, loudness/timbre, pitch, tempo, attack slope, spectral spread, spectral irregularity, key, and musical surprises (see methods and Supplementary Tables S1 and S2 for details about the extracted musical factors). We then applied multiple regression analysis in each of the musical sections, except for the second part of Mussorgsky’s piece, to examine how these distinct musical attributes may account for the continuous self-reports of valence and arousal. The results of this analysis are summarized in Table 2. As expected, even when accounting for other features, inter-subject-tapping-coherence remained a significant factor in positively predicting valence in the examined musical sections. No other factors consistently explained the ongoing fluctuation in reported valence across the different pieces (Table 2a). Fluctuations in reported arousal, on the other hand, were not explained consistently by the inter-subject-tapping-coherence, but were robustly and positively explained in the three pieces by the ongoing variations in tempo and in loudness/timbre factors (Table 2b). This observation nicely replicates previous findings (e.g., Chapin, Jantzen, Kelso, Steinberg, & Large, 2010; Schubert, 2004) and thus supports the analysis approach utilized here.

**Table 2:** Temporal predictability in a wider context – Musical dimensions and the reported experience.


A. VALENCE								

	GLASS (n = 36)		LIGETI, RIC. 1 (n = 37)		LIGETI, RIC. 2 (n = 37)		MUSSORGSKY, PART 1 (n = 34)	
			
	MEAN *B*(SE)	p-VALUE (BOOTSTRAP)	MEAN *B*(SE)	p-VALUE (BOOTSTRAP)	MEAN *B*(SE)	p-VALUE (BOOTSTRAP)	MEAN *B*(SE)	p-VALUE (BOOTSTRAP)

Loudness/ timbre	–0.026 (0.012)	*p* = 0.038	–0.038 (0.018)	***p* = 0.02**	–0.032 (0.009)	***p* < 0.001**	–0.055 (0.015)	***p* < 0.001**

Pitch	–0.008 (0.006)	*p* = 0.28	–0.015 (0.008)	*p* = 0.05	–0.002 (0.012)	*p* = 0.8	0.022 (0.01)	*p* = 0.04

Tempo	–0.017 (0.012)	*p* = 0.17	–0.046 (0.018)	***p* = 0.006**	–0.021 (0.012)	*p* = 0.09	–0.051 (0.015)	***p* < 0.001**

Attack Slope	–0.003 (0.006)	*p* = 0.67	0.031 (0.011)	***p* = 0.02**	0.02 (0.01)	*p* = 0.05	0.001 (0.006)	*p* = 0.88

Spectral Spread	0.072 (0.012)	***p* < 0.001**	–0.025 (0.01)	***p* = 0.006**	–0.008 (0.003)	*p* = 0.06	0.001 (0.005)	*p* = 0.87

Spectral Irregularity	0.022 (0.004)	***p* < 0.001**	0.027 (0.007)	***p* = 0.02**	–0.009 (0.004)	*p* = 0.054	–0.009 (0.005)	*p* = 0.05

Key	0.012 (0.004)	***p* = 0.02**	0.046 (0.019)	***p* = 0.006**	–0.003 (0.003)	*p* = 0.38	–0.012 (0.002)	***p* < 0.002**

Musical Surprises	0.011 (0.003)	***p* = 0.002**	0.003 (0.004)	*p* = 0.38	0.007 (0.003)	*p* = 0.07	0.002 (0.005)	*p* = 0.66

Inter-subject-tapping-coherence	0.037 (0.007)	***p* < 0.0001**	0.034 (0.012)	***p* = 0.003**	0.021 (0.009)	***p* = 0.01**	0.019 (0.005)	***p* < 0.001**

**B. AROUSAL**								

	**GLASS (n = 36)**		**LIGETI, RIC. 1 (n = 37)**		**LIGETI, RIC. 2 (n = 37)**		**MUSSORGSKY, PART 1 (n = 34)**	
			
	**MEAN *B* (SE)**	**p-VALUE (BOOTSTRAP)**	**MEAN *B* (SE)**	**p-VALUE (BOOTSTRAP)**	**MEAN *B* (SE)**	**p-VALUE (BOOTSTRAP)**	**MEAN *B* (SE)**	**p-VALUE (BOOTSTRAP)**

Loudness/ timbre	0.119 (0.016)	***p* < 0.001**	0.111 (0.015)	***p* < 0.001**	0.076 (0.01)	***p* < 0.001**	0.122 (0.015)	***p* < 0.001**

Pitch	0.04 (0.009)	***p* < 0.001**	0.03 (0.01)	***p* < 0.001**	0.046 (0.012)	***p* < 0.001**	0.017 (0.01)	*p* = 0.094

Tempo	0.082 (0.015)	***p* < 0.001**	0.105 (0.011)	***p* < 0.001**	0.113 (0.017)	***p* < 0.001**	0.098 (0.014)	***p* < 0.001**

Attack Slope	0.006 (0.006)	*p* = 0.48	0.002 (0.007)	*p* = 0.82	–0.014 (0.011)	*p* = 0.26	–0.007 (0.003)	*p* = 0.17

Spectral Spread	–0.022 (0.01)	***p* = 0.023**	–0.019 (0.007)	***p* = 0.02**	0.013 (0.003)	***p* = 0.013**	–0.027 (0.006)	***p* < 0.001**

Spectral Irregularity	–0.005 (0.004)	*p* = 0.25	0 (0.008)	*p* = 0.99	–0.009 (0.006)	*p* = 0.15	–0.011 (0.005)	***p* = 0.02**

Key	0.011 (0.004)	*p* = 0.07	0.034 (0.013)	***p* = 0.01**	0.016 (0.004)	***p* = 0.0001**	0.011 (0.003)	***p* = 0.002**

Musical Surprises	0.005 (0.003)	*p* = 0.25	0.008 (0.002)	*p* = 0.026	0.003 (0.003)	*p* = 0.51	0.003 (0.004)	*p* = 0.49

Inter-subject-tapping-coherence	0.001 (0.008)	*p* = 0.97	–0.009 (0.009)	*p* = 0.41	0.016 (0.01)	*p* = 0.11	0 (0.005)	*p* = 0.96


*Note A:* Averages of regression coefficients for the nine musical dimensions ±1 SEM for explaining continuous valence ratings are depicted per index of reported experience along with the level of statistical significance. Effects reaching statistical significance of p < .05 after False Discovery Rate correction for multiple comparisons are highlighted in light grey. Musical factors that show consistent effects across sections are highlighted in dark grey. *Note B:* Averages of regression coefficients for the nine musical dimensions ±1 SEM for explaining continuous arousal ratings are depicted per index of reported experience along with the level of statistical significance. Effects reaching statistical significance of p < .05 after False Discovery Rate correction for multiple comparisons are highlighted in light grey. Musical factors that show consistent effects across sections are highlighted in dark grey.

### Generalization of Findings using a Large Online Database (DEAM)

**Figure 3:**
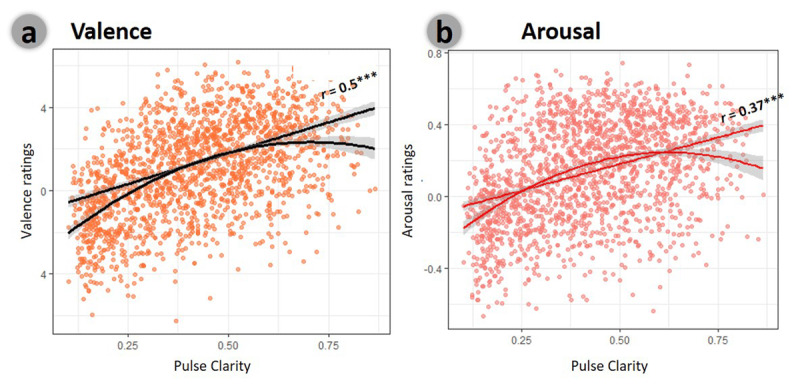
**DEAM database (Aljanaki et al., 2017) – support for the association between temporal predictability and music-induced emotions.** *Overall ratings (at the level of the entire song)*: Linear and quadratic regressions of overall pulse clarity for ratings of: **a.** valence and **b.** arousal. Markers represent the mean rating for each of 1780 songs taken from the DEAM database as a function of its overall pulse clarity. Lines represent the regression fit across songs.

Motivated to generalize our finding into a larger and more diverse dataset, we turned to the DEAM database (Aljanaki et al., 2017), which includes continuous annotations of valence and arousal of about 1800 songs. We tested the prediction that within this diverse pool of musical materials, the valence ratings will co-vary across songs with temporal predictability, which was indexed here for practical reasons using the automatically extracted index of pulse clarity (see methods for details). We first inspected how the valence ratings, averaged across the entire piece, vary as a function of pulse clarity levels across the different songs. As expected, there was a strong positive linear association between pulse clarity levels and the mean valence ratings across the different songs (*r* =.50; Figure 3a). This finding indicates that more temporally regular songs were rated overall as more pleasant. This relationship was significantly higher than the one observed between pulse clarity and arousal (*r* = .37; Figure 3b; Fisher Z test for two dependent correlations; *Z_i_* = 6.9; *p* < .001). We further tested if such relationship between mean valence and pulse clarity follows the form of a u-shaped curve as anticipated from theories of aesthetic appreciation about the relation between the complexity or novelty of a stimulus and its hedonic tone (Berlyne, 1971). For that, we applied curvilinear regression analysis to predict valence with the first and second polynomial degrees of pulse clarity. Indeed, the model explained a statistically significant and substantial proportion of the variance (adj. R^2^ = 0.28, *F*(2, 1773) = 340.66, *p* < .001). Within this model, the linear (*β* = 4.82, 95% CI [4.44, 5.20], *t*(1773) = 24.76, *p* < .001) and quadratic effects of pulse clarity were statistically significant (*β* = –1.61, 95% CI [–1.99, –1.23], *t*(1773) = –8.27, *p* < .001; Figure 3a). This model, which combined both the linear and quadratic terms, fit the data significantly better than a model that contained only linear terms [comparison: *χ*²(1) = 612.85 p < .001]. These results confirm and extend our findings of a consistent and robust relation, over a large variety of songs, between music-induced pleasantness and the extent of temporal predictability; captured here using the automatically extracted measure of pule clarity. Similar findings, although with a moderate proportion of explained variance (adj. R^2^ = 0.17, *F*(2, 1773) = 180.12, *p* < .001), were evident for the arousal dimension, with statistically significant linear (*β* = 4.37, 95% CI [3.87, 4.87], *t*(1773) = 17.15, *p* < .001) and quadratic effects of pulse clarity (β = –2.07, 95% CI [–2.57, –1.57], *t*(1773) = –8.14, *p* < .001, comparison with linear fit, χ²(1) = 293.99 p < .001; Figure 3b).

## Discussion

Music, often referred to as ‘the language of emotion,’ is a multifaceted phenomenon that elicits complex emotional responses influenced by a plethora of variables as they unfold in time. Among these features, important factors known to influence the emotional response are the ongoing processes of anticipation, prediction, error correction, and reward associated with these processes. Yet, measuring aspects of behavior that continuously index the predicted musical structure in a naturally unfolding musical piece is not trivial. In the current study, we focused on this fascinating aspect by examining how music-induced emotions change as a function of temporal predictability as the music unfolds. Temporal predictions rely on the percept of the musical beat – an internal construct rather than a mere reflection of the acoustic events. Moreover, temporal predictions are embedded in auditory-motor integration circuits (Cannon & Patel, 2021). We therefore turned to beat-tapping data of musically trained listeners as the best source for capturing such fluctuations in temporal predictions as the music unfolds. We relied on the notion that across a group of different tappers, moments that are more predictable will be associated with more incidents of well-synchronized taps. Using this measure, we found that more temporally predictable moments are experienced as more pleasant, even when controlling for additional musical features. These observations were further supported and generalized using an automatically extracted feature of pulse clarity, applied to a large database that contains annotations of valence and arousal in response to music.

Our findings are in accordance with several lines of studies. One line of evidence comes from studies that used a large set of automatically extracted musical features to predict subjective ratings of music-induced emotions. Among these features, pulse clarity (Lartillot, Eerola, et al., 2008) – used as a proxy for temporal predictions – was found to be associated with changes in valence and arousal (Trost et al., 2015), or to be the second most effective feature in predicting global valence (Eerola, 2011), though in a genre-specific way (most prominent in pop music). In the current study, this automatically extracted index of pulse clarity, which partly correlated with our index of inter-subject-tapping-coherence, was used to demonstrate that the link between temporal predictions and valence may extend to other musical styles within a larger data pool of 1780 songs. Another line of evidence comes from studies that focused on the phenomenon of groove – the pleasurable urge to move to the beat of music (Janata, Tomic, & Haberman, 2012; Witek et al., 2014). In particular, Janata and colleagues (2012) showed that the quality of sensory-motor synchronization to a beat positively correlated with the experience of being in the groove and therefore enjoyment. Adding to this line of research, Witek and colleagues (2014) reported that the sense of groove and feelings of pleasure depended on the complexity of syncopation in an inverted u-shaped fashion, with an optimal “sweet spot” that balances between complexity and simplicity (Matthews et al., 2019; Stupacher et al., 2022). Thus, consistent with our findings, these studies suggest that the extent of ability to match movements to a perceived musical pulse has hedonic quality. This may provide support to the notion that the alignment of one’s bodily rhythms, motor actions or attention, to a periodically perceived pulse plays a key role in explaining the emotions induced by listening to music (Juslin, 2013; Juslin et al., 2010; Trost & Vuilleumier, 2013). Our study extends these findings by revealing the link between pleasantness, which relates to the concept of valence, and beat strength in “non-groovy” musical contexts and is evident not only globally, but also locally, on a moment to moment basis as music unfolds. Finally, a third line of evidence comes from a study that addressed the question of temporal expectancies in music by showing that music-induced emotions can be predicted from descriptors of onset predictability that were derived from models that rely on statistical learning (Egermann et al., 2013; Gold, Pearce, et al., 2019; Sauvé et al., 2018). Specifically, in this study, Sauvé and colleagues used the information dynamics of music (IDyOM) model to depict the extent to which each note onset is predictable by estimating the probability from the statistics of their occurrence as they evolve in the song (short-term) and more globally in music of certain styles. Using this model, they found that the onset predictability explained both arousal and valence dimensions of the emotional response to music, being higher for more predictable pieces. Yet, the reliance on the analysis of pre-selected corpuses of music, mainly western folk songs in MIDI format, limits the use of this approach when considering the responses to much more complex musical pieces with expressive performance fluctuations in tempo, microtiming, intensity, timbre, articulation, etc.

Since temporal predictability was operationally defined here as the extent of inter-subject-tapping-coherence, it is possible that its hedonic value can be further associated to its social value as it represents the potential of synchronizing our movements with others through temporal prediction, an act that may have hedonic value of its own (Overy & Molnar-Szakacs, 2009). Along that line, evolutionary psychologists have long assigned an important role to rhythmic engagement and music as a means for social bonding, cohesion and possibly for regulating the groups’ affective state (Cross, 2014; Dunbar, 2012; McNeill, 1997; Tarr, Launay, & Dunbar, 2014). Indeed, musical pleasure has been shown to be closely tied to emotions that are associated with the sense of belonging (Saarikallio, Maksimainen, & Randall, 2019). This suggestion resonates with our finding that temporal predictability was associated with valence in excerpts that were characterized by tempi within the range of salient beat perception (i.e., 80 to 160 Bpm; Figure S1).

The one exception for our findings was in the second slower part of Mussorgky’s piece. In this section of the piece, the relationship between valence and predictability measures behaved in an opposing fashion – showing a negative correlation between temporal predictability and pleasantness. It has been established that accuracy of tapping is significantly reduced in tempi slower than 80 Bpm, as that found in slower part of Mussorgky’s piece (Møller, Stupacher, Celma-Miralles, & Vuust, 2021). This suggests that temporal predictions in such pieces cannot be as pleasing as in the optimal range of 80 to 160 Bpm. Slower songs or sections may rely more on other musical features and on other psychological mechanisms such as emotional contagion (Juslin, 2013). In fact, slow movements characterized by a lyrical melody as found in the last section of Mussorgsky, require from players extra expressivity to convey the quality of emotional speech. Hence, despite pointing to a general mechanism that seems to contribute to the hedonic response to music, we further highlight that the association is weakened or even reversed under some conditions. However, we suggest to examine in further studies whether this reversal is influenced more by the musical genre as proposed by Eerola (2011) or rather by the tempo of the piece.

### Limitations

In this study, we focused on explaining the responses to naturalistic and relatively long pieces and used experts-based annotations for indexing temporal predictability – two experimental decisions that limited the number of musical materials used. The use of a small number of musical pieces in our study therefore limits our ability to generalize our conclusions to other musical contexts and genres. To address this shortcoming, we focused on effects that were consistent across highly distinct musical pieces in terms of their tonal and temporal design and supported them using large database of songs that contains annotations of music-induced emotions (Aljanaki et al., 2017). Further, our ability to generalize the conclusions is somewhat limited by the relatively modest sample size of participants we used. Future studies should replicate these findings while incorporating another set of musical materials. It should also be noted that the tapping data and the subjective ratings were obtained from completely different groups. Future studies could aim to gather both emotional ratings and tapping data from the same participants (both musically trained and untrained). In that case, individual differences in tapping synchronization to the beat may be further used to predict the level of music-induced pleasantness.

It is also important to note that while this study highlighted the role of temporal predictability in music-induced emotions, other musical features and variables, such as loudness, timbre, pitch height, harmonic and melodic complexity also play a significant role in shaping the emotional experience of music (Chapin et al., 2010; Coutinho & Cangelosi, 2011; Eerola, 2011; Eerola et al., 2009; Gabrielsson, 2014; Stupacher, Hove, & Janata, 2016), and were found to correlate with this experience in our study as well (Table 2). This multi-level account also suggests that there may be an interaction between the different features (Granot & Eitan, 2011). Therefore, further investigation into how the interaction between different musical features may affect the ongoing emotional experience, as demonstrated in previous studies on groove using systematic manipulations of features such as harmonic or rhythmic complexity (Matthews et al., 2019), or bass frequency and attack time (Stupacher et al., 2016), would be intriguing. Another important consideration for future research is whether musical pleasure and music-induced emotional valence, as measured in this study, are equivalent constructs that are similarly affected by temporal predictability (Goupil & Aucouturier, 2019). For example, studies have shown that pharmacological dopamine manipulation can impact reported pleasure levels but not valence and arousal levels (Ferreri et al., 2019), and that individuals may derive enjoyment and pleasure from sad or negatively valenced songs (Sachs, Damasio, & Habibi, 2015), highlighting the need for further investigation into the complexities of music-induced emotions (for example, Keller & Schubert, 2011).

Finally, using naturalistic music enhances the ecological validity of the investigation and allows for uncovering the temporal unfolding of music-induced emotions in relation to temporal predictability. However, it also introduces additional variables that could account for such experiences. Although we controlled for some of these variables in the current investigation using the multiple regression analyses, future studies should complement our findings using a controlled set of stimuli that vary solely in terms of rhythm, while keeping all other variables constant. Similar studies investigating groove have yielded findings that align with our current work (Matthews et al., 2019; Stupacher et al., 2022; Witek et al., 2014).

## Conclusions and Theoretical Perspectives

The findings of this study may be interpreted in light of the theoretical perspective of predictive coding (Koelsch, Vuust, & Friston, 2018; Witek et al., 2014). In a nutshell, the theory of predictive coding asserts that the brain continuously generates models based on current contexts and prior knowledge in order to predict incoming input. As such, neural computations are mainly tuned into minimizing prediction error – the difference between an internal model and the input. Recently, this framework has been adopted to explain music listening as an active process that involves the constant generation of predictions and their subsequent violation (Koelsch et al., 2018). Such a process includes both the prediction of content and the precision by which this content can be predicted. This layer of precision has been formulated as an important factor that filters our responses to (content) prediction violations, such that we respond more vigorously to “predictably surprising” events and ignore imprecise prediction errors. Bearing these notions in mind, it can be suggested that the predictability of the next note may be an important factor for the precision layer as it allows generating a predictive model of the timing of subsequent musical events, possibly via a neural resonance mechanism (Large, Fink, & Kelso, 2002). Such enhanced temporal predictability may augment the listeners’ responses to- and potential learning benefits from- predictably surprising events. The latter musical prediction errors have been conceptualized as important drivers of musical pleasure (Salimpoor et al., 2015), and have been recently shown to engage major nodes of the brain’s reward system (Gold, Mas-Herrero, et al., 2019; Shany et al., 2019) and of the limbic network (Cheung et al., 2019). Extending to other domains, our findings may also be relevant to other affective processes – suggesting that regularities and their violations provide invaluable basic affective codes.

## Data Accessibility Statement

The tapping based musical annotations and the additional features used in this study are available in the supplementary materials. We would like to include this data file as a supplementary material. See details below. The individual rating data will be provided upon request from the author, depending upon IRB approval.

## Additional Files

The additional files for this article can be found as follows:

10.5334/joc.286.s1 Figure S1. Tempo per musical section.

10.5334/joc.286.s2 Figure S2. Distinct tapping patterns along the Glass piece.

10.5334/joc.286.s3 Table S1. List of the extracted musical features.

10.5334/joc.286.s4 Table S2. Summary of musical factor loadings.

10.5334/joc.286.s5 Table S3. Association between temporal predictability, calculated using the entire group of tappers, and behavioral responses to music: summary of correlation analyses and paired t-tests.

10.5334/joc.286.s6 Supplementary materials. Supplementary data: Musical features and tapping based annotations from the current study.
