# Supplementary material for: Feeling the Beat: Temporal Predictability is Associated with Ongoing Changes in Music-Induced Pleasantness
Source: J Cogn. 2023 Jul 4;6(1):34. doi: 10.5334/joc.286 (PMC10348017; doi:10.5334/joc.286)
Supplement: Figure S1. — Tempo per musical section. [file joc-6-1-286-s1.pdf]

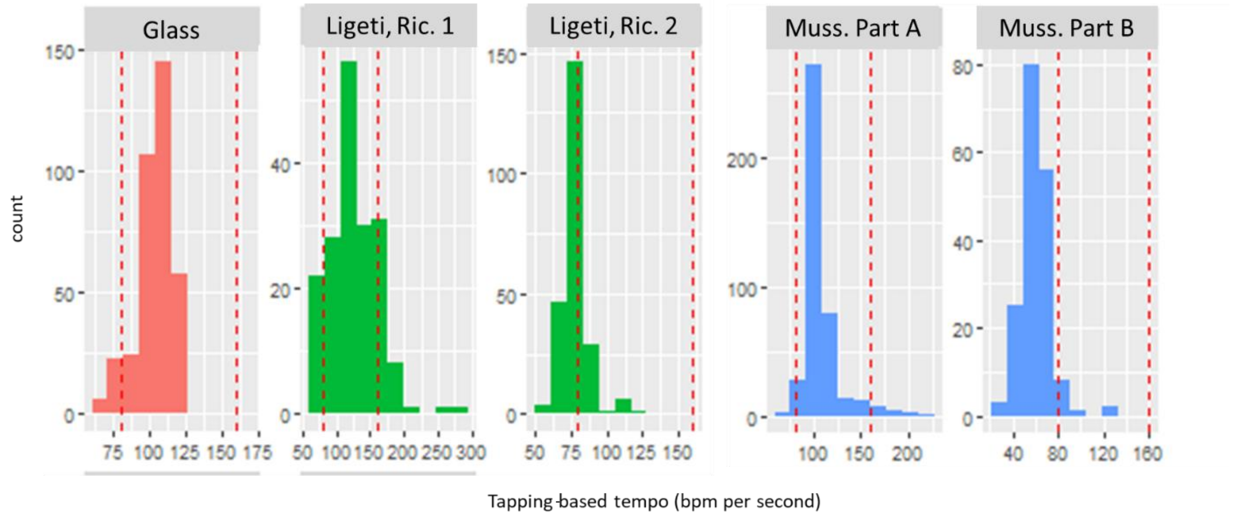


**Figure S1. Tempo per musical section.** Histogram representing the frequency of tapping-based tempo in each musical section. Tempo was indexed in each second as beats per minute (bpm) based on the following formula: (1 / Inter Tap Interval) * 60. The dashed red lines mark bpm range that is optimal for salient beat perception, between 80 and 160.
