# Supplementary material for: Feeling the Beat: Temporal Predictability is Associated with Ongoing Changes in Music-Induced Pleasantness
Source: J Cogn. 2023 Jul 4;6(1):34. doi: 10.5334/joc.286 (PMC10348017; doi:10.5334/joc.286)
Supplement: Figure S2. — Distinct tapping patterns along the Glass piece. [file joc-6-1-286-s2.pdf]

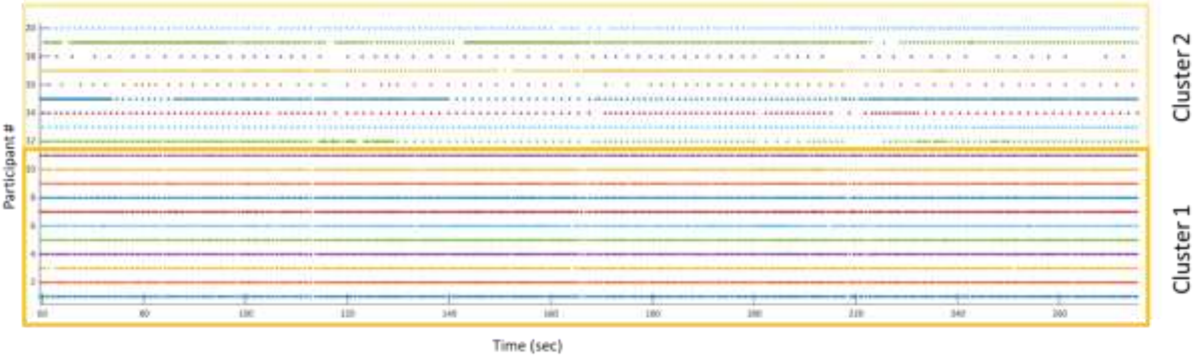

Figure S2. **Distinct tapping patterns along the Glass piece**. Each row corresponds to the tapping pattern of a different musician. The tapping data were clustered into two clusters, which are highlighted by yellow rectangles. The lower rectangle denotes the first cluster of particpants, who maintained a constant level despite the metrical changes from triple to duple meter throughout the piece. In contrast, the second cluster of participants (upper rectangle), exhibited changes in the inter-tap interval at the chosen tactus level in different parts of the piece. We selected the more consistent tapping pattern of the first cluster (Cluster 1) for our analyses. Notably, our results remain robust even when considering the entire dataset (see Table S3).
