# Supplementary material for: Feeling the Beat: Temporal Predictability is Associated with Ongoing Changes in Music-Induced Pleasantness
Source: J Cogn. 2023 Jul 4;6(1):34. doi: 10.5334/joc.286 (PMC10348017; doi:10.5334/joc.286)
Supplement: Table S1. — List of the extracted musical features. [file joc-6-1-286-s3.pdf]

Table S1. List of the extracted musical features

| Musical feature name | Description | Toolbox | Function used | Extracted based on |
|---|---|---|---|---|
| **Spectral Centroid** | Center of mass of the spectrum | MIR toolbox (*1*) | mircentroid.m & mirspectrum.m | sound file |
| **Brightness** | High frequency energy | MIR toolbox | mirbrightness.m | sound file |
| **Dynamic Loudness** | Perceived loudness, based on Chalupper and Fastl (*2*) | PsySound3 (*3*) | Dynamic Loudness | sound file |
| **Roughness** | Estimated based on computing the average ratio between all possible pairs of spectral peaks to index the beating sensation that arises when two frequencies are proximal. | MIR toolbox | mirroughness.m | sound file |
| **Spectral Flux** | Distance between the spectrums of successive frames. | MIR toolbox | mirflux.m & mirspectrum.m | Sound file |
| **Chromagram** | Energy distribution along the pitch classes | MIR toolbox | mirchromagram.m | Sound file |
| **Pitch: Autocorrelation, peak height** | height of the largest non-zero lag autocorrelation peak | Psysound3 | Auto-correlation, peak height | Sound file |
| **Attack Time** | Temporal duration of attack phases. Attack: the time it takes the sound to reach a certain threshold | MIR toolbox | mirattacktime.m | Sound |
| **Attack Slope** | Average slope of attack phases | MIR toolbox | mirattackslope.m | sound |
| **Spectral spread** | The standard deviation | MIR | mirspread.m | Sound |

| | | | | |
|---|---|---|---|---|
| | of the spectrum | toolbox | | |
| **Spectral irregularity** | The degree of spectral variation between successive peaks | MIR toolbox | Mirirregularity.m | sound file |
| **Key** | Estimation of the of tonal centers | MIR toolbox | Mirkey.m | sound file |
| **Event density** | The average frequency of detected sound events | MIR toolbox | Mireventdensity.m | sound file |
| **Pulse clarity** | Estimation of the rhythmic clarity based on the maximal autocorrelation in the detected sound events | MIR toolbox | Mirpulseclarity.m | sound file |
| **Inter-subject-tap-coherence** | Estimation of how well the timing of the next beat is predicted based on the temporal alignment of taps to the beat; determined by a tapping experiment. See main text | In house analysis | Number of aligned taps across experts within a narrow windows of 100 ms | Expert's annotation: tapping |
| **Tempo: frequency of taps (Beats per minute)** | Estimation of the momentary tempo based on the inter-tap interval determined by a tapping experiment. See main text. | In house analysis | 1 / median (inter-tap interval) *60 | Expert's annotation: tapping |
| **Musical surprises** | Annotation of moments of expectancy violations based on experts' indications. See (*4*) | In house analysis | Number of experts indicating a surprising moment | Expert's annotation: indication of surprising moments |

*Note: all of the features were extracted in frames and were brought to a sampling resolution of 1Hz.

**References:**


O. Lartillot, P. Toiviainen, A Matlab Toolbox for Musical Feature Extraction from Audio, 8 (2007).

2. J. Chalupper, H. Fastl, Dynamic Loudness Model (DLM) for Normal and Hearing-Impaired Listeners. *Acta Acust. United Acust.* **88**, 378–386 (2002).

3. D. Cabrera, S. Ferguson, E. Schubert, “Psysound3”: Software for Acoustical and Psychoacoustical Analysis of Sound Recordings (2007) (available at https://smartech.gatech.edu/handle/1853/49969).

4. O. Shany, N. Singer, B. P. Gold, N. Jacoby, R. Tarrasch, T. Hendler, R. Granot, Surprise-related activation in the nucleus accumbens interacts with music-induced pleasantness. *Soc. Cogn. Affect. Neurosci.* **14**, 459–470 (2019).
