# Supplementary material for: Feeling the Beat: Temporal Predictability is Associated with Ongoing Changes in Music-Induced Pleasantness
Source: J Cogn. 2023 Jul 4;6(1):34. doi: 10.5334/joc.286 (PMC10348017; doi:10.5334/joc.286)
Supplement: Table S2. — Summary of musical factor loadings. [file joc-6-1-286-s4.pdf]

Table S2. Summary of musical factor loadings

**Total explained variance = 89.58%**	Component								
	1 Loudness/ timbre (27.32%)	2 Pitch (10.53%)	3 Tempo (9.99%)	4 Attack Slope (7.64%)	5 Spectral Spread (7.31%)	6 Spectral Irregularity (6.79%)	7 Key (6.75%)	8 Musical Surprises (6.75%)	9 Inter-subject-tap-coherence (6.5%)
Spectral Centroid	.893	.134	.152	.221	.124	.007	.026	.060	.139
Brightness	.857	-.052	.194	.191	-.045	-.085	-.002	.102	.141
Dynamic Loudness	.856	.022	.129	.127	.193	.078	.001	-.014	.083
Roughness	.804	-.302	.064	-.272	.035	-.080	.075	-.047	.038
Spectral Flux	.744	.056	.257	.251	.339	.058	.038	-.025	.025
Chromagram	-.101	-.896	-.002	.080	.031	-.083	-.091	.036	.175
Pitch: autocorrelation peak height	-.174	.774	.056	.334	.060	.105	-.020	.051	.211
Tempo: frequency of taps*	.214	-.035	.908	.040	.129	-.038	.043	-.006	.134
Attack time	.552	.145	.694	-.059	-.070	-.035	.071	.038	.070
Attack slope	.322	.121	.000	.882	.048	.073	.008	-.012	-.026
Spectral Spread	-.253	-.006	-.072	-.039	-.927	-.069	-.080	.002	-.158
Spectral Irregularity	-.016	.144	-.049	.069	.065	.981	.032	-.014	.038
Tonal center (Key)	.047	.064	.067	.005	.072	.032	.985	.010	.090
Musical Surprises*	.052	.002	.011	-.006	-.003	-.013	.009	.992	-.058
Inter-subject-tap-coherence*	.267	-.030	.173	-.020	.169	.045	.111	-.075	.889

Note: Nine orthogonal principal components, representing the musical features used in the regression analysis for examining the effect of temporal predictability when accounting for additional musical features. Factor loadings for each component, as well as the explained variance of each component are depicted. The principal components were extracted across Ligeti, Glass, and Mussorgsky pieces. Asterisks denote the source for musical annotation as follows: *based on the ratings of 20 experts; none: based on the sound files.
