# Supplementary material for: Feeling the Beat: Temporal Predictability is Associated with Ongoing Changes in Music-Induced Pleasantness
Source: J Cogn. 2023 Jul 4;6(1):34. doi: 10.5334/joc.286 (PMC10348017; doi:10.5334/joc.286)
Supplement: Table S3. — Association between temporal predictability, calculated using the entire group of tappers, and behavioral responses to music: summary of correlation analyses and paired t-tests. [file joc-6-1-286-s5.pdf]

Table S3. Association between temporal predictability, calculated using the entire group of tappers, and behavioral responses to music: summary of correlation analyses and paired t-tests

**a. Valence**

| | Mean *r* (SEM) | p- value (bootstrap) | Compare high vs. low |
|---|---|---|---|
| **Glass** (*n*=36) | .12 (.03) | $<.001^{***}$ | $t(35) = 3.85$; $p<.001^{***}$ |
| **Ligeti, Ric. 1** (*n*=37) | .23 (.07) | $<.001^{***}$ | $t(36) = 3.70$ $p<.001^{***}$ |
| **Ligeti, Ric. 2** (n=37) | .04 (.02) | $.056^{+}$ | $t(36) = 2.27$; $p = .0294^{*}$ |
| **Mussorgsky, Mov. 1** (*n* = 34) | .07 (.01) | $<.001^{***}$ | $t(33) = 2.53$; $p = .02^{*}$ |
| **Mussorgsky, Mov. 2** (*n* = 34) | -.24 (.04) | $<.001^{***}$ | $t(33) = -4.68$; $P<.001^{***}$ |

**b. Arousal**

| | Mean r (SEM) | p- value (bootstrap) | Compare high vs. low |
|---|---|---|---|
| **Glass** (*n*=36) | -.02 (.03) | .51 | $t(35) = 0.4$; $p = .68$ |
| **Ligeti, Ric. 1** (*n*=37) | -.06 (.05) | .34 | $t(36) = -0.81$; $p = .42$ |
| **Ligeti, Ric. 2** (n=37) | -.05 (.02) | .005 | $t(36) = -2.41$; $p = .0214^{*}$ |
| **Mussorgsky, Mov. 1** (*n* = 34) | -.02 (.02) | .18 | $t(33) = -.69$; $p =$ .49 |
| **Mussorgsky, Mov. 2** (*n* = 34) | .35 (.03) | $<.001^{***}$ | $t(33) = 7.95$; $P<.001^{***}$ |

**Note**: To examine the effects of the methodological decision of selecting consistently tapping musicians for annotating the inter-subject tapping coherence, we conducted an additional analysis using a measure of inter-subject tapping coherence that was calculated with all available datasets without clustering beforehand. The results of this validation analysis, which are presented in this table, show a consistent pattern of results similar to the original analysis. Averages and S.E.M of correlation coefficients between inter-subject tapping coherence, without removing any group in advanced, and the ongoing fluctuations in reported a. valence or b. arousal per musical excerpt. The statistical significance, which was estimated using a phase randomization bootstrapping approach, is further indicated. T-values representing the result of a paired sample t-test for the comparison between the average rating during moments of high vs. low moments temporal predictability are further provided. Effects corrected for multiple comparisons are highlighted in gray (FDR-corrected, $q(FDR) < .05$; + marginal significance, $q(FDR) < .06$).

**Abbreviations**: Synch. = synchronization
